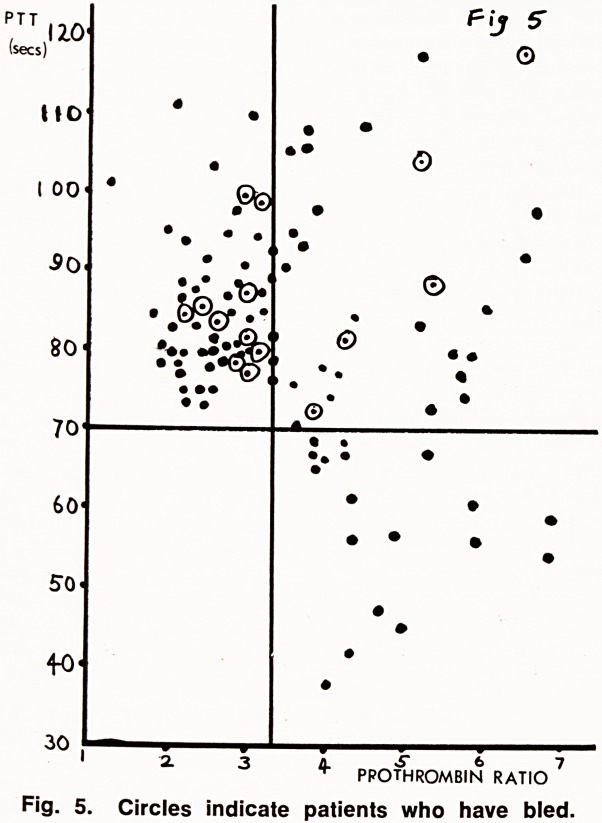# Problems of Anticoagulant Control—II, Which Test?

**Published:** 1971-10

**Authors:** T. J. Hamblin

**Affiliations:** Dept. of Pathology, Southmead Hospital, Bristol


### Problems of Anticoagulant Control II Which Test ?

**T. J. Hamblin, M.B., Ch.B., M.R.C.P.**
Dept. of Pathology, Southmead Hospital, Bristol

"It is a sad reflection on the limitations of clinical trials that 20 years after the introduction of oral anti-coagulants their place in the long term management of coronary artery disease is still disputed" . . . B.M.J. leader February 1970 ([2]).

It is more of a reflection on the limitations of the tests used to control anticoagulants. For example, after the publication of the most recent M.R.C. report on the use of anticoagulants after cardiac infarction ([10]), it became apparent that three years work in eleven centres involving nearly 1500 patients was invalidated because the level of anticoagulant control —although that originally advocated by the manufacturers of the thromboplastin used—had since been shown to result in homeopathic dosages well below the therapeutic range ([11]).

The function of any test used for anticoagulant control is to ensure that enough of the drug is given to prevent thrombotic episodes, yet not enough to cause haemorrhagic complications. In practice no one has determined just how much is necessary to prevent thrombosis, and the usual procedure is to give the maximum dose compatible with safety ([1]). The test must therefore be able to predict bleeding.

When blood escapes from a damaged vessel clotting is a relatively simple matter (Fig. 1); on the other hand clotting initiated within a vessel, because of stasis, or damage to the intima, is a rather more involved affair (Fig. 2).

Oral anticoagulants act by depressing the levels of Factors VII, IX, X, and II in the blood in that order. Factor VII is more greatly affected than the others especially at the start of treatment, and is concerned only in the extrinsic clotting system. Factor IX is concerned only in the intrinsic system while the other two are involved in both.

### THE ONE STAGE PROTHROMBIN TIME

This is the most widely used test for anticoagulant control, and was introduced by Quick in 1935 ([8]). In this test a brain extract is used as a source of tissue activator, and is added to citrated plasma which is then recalcified. The time taken for the plasma to clot is then measured. The result is usually expressed as a ratio of the test time to that of a normal control. This test is therefore a measure of the effectiveness of the extrinsic clotting system (fig. 1), and in patients on oral anticoagulants is most affected by Factor VII levels ([9]).

Unfortunately the ratio thus obtained varies with the type of brain extract used. There are many different commercial extracts on the market, and in addition many laboratories make their own. The result of all this free enterprise is that the prothrombin ratio is not comparable from laboratory to laboratory. Poller ([6]), recognising this, has introduced a reference reagent and, in future, patients on anticoagulants may be able to travel around the country in relative safety.

Even when this objection to the prothrombin ratio is removed, it remains a test of the extrinsic clotting system. The purpose of the treatment is to reduce coagulation within blood vessels and therefore an effect on the intrinsic system is desired. Any benefit that accrues by depressing the extrinsic system to a given level is fortuitous, and depends on the hope that the intrinsic system will be similarly affected. Although this sometimes happens, such a hope cannot be relied upon (fig. 3).

It would be reasonable to control anticoagulants using a test of the extrinsic system if defects in this system were the cause of the bleeding due to anti-coagulant overdose; however. it has long been recognised that there is no hard and fast relationship between the prothrombin ratio and the tendency to bleed ([5]). This bleeding is typically haematuria and spontaneous bruising and this latter especially resembles the bleeding seen in thrombocytopenia and disorders of the platelet function, rather more than that seen in deficiencies of clotting factors such as haemophilia or Christmas disease. In this respect it is very interesting that Poller ([7]) has found that in patients taking anticoagulant drugs, defects in the intrinsic system affect platelet function in a way that defects in the extrinsic system do not.

It would seem therefore that on both counts—efficiency of anti-coagulation and freedom from haemorrhage—it is better to monitor the intrinsic system than the extrinsic system.

### THROMBOTEST

It seemed likely at one time that the Thrombotest would give its users the best of both worlds, in that it was claimed to detect deficiencies in all the four factors depressed by oral anticoagulants. Moreover the test can be performed on capillary blood, and enjoyed a vogue particularly with the organisers of controlled trials, for whom it provided a method which was comparable from centre to centre. However Denson ([3]) has shown that a Factor IX level of 1%, sufficient to cause torrential haemorrhage in a case of Christmas disease, only lowers the Thrombotest to 80%, which is within the normal range. In effect, therefore, Thrombotest is an expensive, albeit quick and convenient way of assessing the extrinsic system.

Fig. 1. The Extrinsic Clotting System

Fig. 2. The Intrinsic Clotting System

Fig. 3.

Fig. 4. Activation of Factor XII and Release of Phospholipid from Platelets are time consuming steps.

### ACTIVATED PARTIAL THROMBOPLASTIN TIME

The whole blood clotting time is too long and too variable to be used to control anticoagulants. The duration and variation are due to two steps which are both time consuming and unpredictably so (Fig. 4). These are the activation of Factor XII, and the release of phospholipid from platelets. The activated partial thromboplastin time (PTT) on the other hand is reliable and reproducible, and much shorter, because Factor XII is activated maximally and rapidly by Kaolin, and an extrinsic source of phospholipid is used.

As a test of the intrinsic system it should be better at predicting bleeding episodes, and should allow a more effective treatment of thrombosis. No information is available on the second point, but Eastham ([4]) using a mechanised version of this test with Bentonite for activation of Factor XII, and soya beans as a source of phospholipid, has compared it with the prothrombin ratio as a method of predicting bleeding in 103 out-patients on long term anti-coagulants. In 21 bleeding episodes in the course of one year, the PTT was greater than 70 seconds in every case, whereas the prothrombin ratio was within the therapeutic range in all but eight cases.

We have looked at the same two tests in a slightly different way, illustrated in Fig. 5. In a three month period, 113 of our patients had results greater than the upper limit of safety with either the prothrombin ratio or the PTT. Of these, 95 had a prolonged PTT and 46 a high prothrombin ratio. There were 15 bleeding episodes (ringed), all of which were associated with a prolonged PTT, but only 5 of which had a high prothrombin ratio.

**Fig. 5. Circles indicate patients who have bled.**

### SUMMARY

In both Eastham's trial and our own the PTT was superior to the prothrombin ratio in predicting bleeding complications in anticoagulated patients. There is no information as to whether patients controlled by PTT have a smaller incidence of thromboembolism than those controlled by prothrombin ratio. However the PTT is unaffected by the initial precipitous fall in Factor VII which seems never to be accompanied by haemorrhage, and which in patients controlled by prothrombin ratio may lead to an under-anticoagulation at a time when danger of thromboembolism is at its greatest.

### REFERENCES

1. Biggs, R. & MacFarlane, R. G. (1962). *Human Blood Coagulation and its Disorders.* 3rd Edition Blackwell Scientific Publications (Oxford).

2. British Medical Journal leader (1970), *Long term Anticoagulant Treatment after Myocardial Infarction,* British Medical Journal **i** 514.

3. Denson, K. W. (1961), *Levels of Blood-Coagulation factors during Anticoagulant Therapy with Phenindione,* British Medical Journal **i** 1204.

4. Eastham, R. D. (1968), *Improved control of long term anticoagulant therapy,* British Medical Journal **ii** 337.

5. Hunter, R. B., & Walker, W. (1954), *Anticoagulant therapy in Myocardial Infarction,* British Medical Journal **ii** 197.

6. Poller, L. (1964), *Standardisation of Anticoagulant treatment: the Manchester Regional Thromboplastin Scheme.* British Medical Journal, **ii** 565.

7. Poller, L., Thomson, J. M., & Priest, C. M. (1969), *Coumarin therapy and Platelet aggregation.* British Medical Journal, **i** 474.

8. Quick, A. J. (1935), *The Prothrombin in Haemophilia and in Obstructive Jaundice.* Journal Biological Chemistry, **109** lxxiii.

9. Quick, A. J. (1961), *Phenindione and Blood Coagulation Factors, British Medical Journal* **ii** 177.

10. Report of Working party on Anticoagulant Therapy to Medical Research Council (1969), *Assessment of short term anticoagulant administration after cardiac infarction.* British Medical Journal **i** 335.

11. Sevitt, S., & Innes, D. (1964), *Prothrombin Time and Thrombotest in Injured patients on prophylactic anticoagulant therapy.* Lancet **i** 124.

## Figures and Tables

**Fig. 1. f1:**
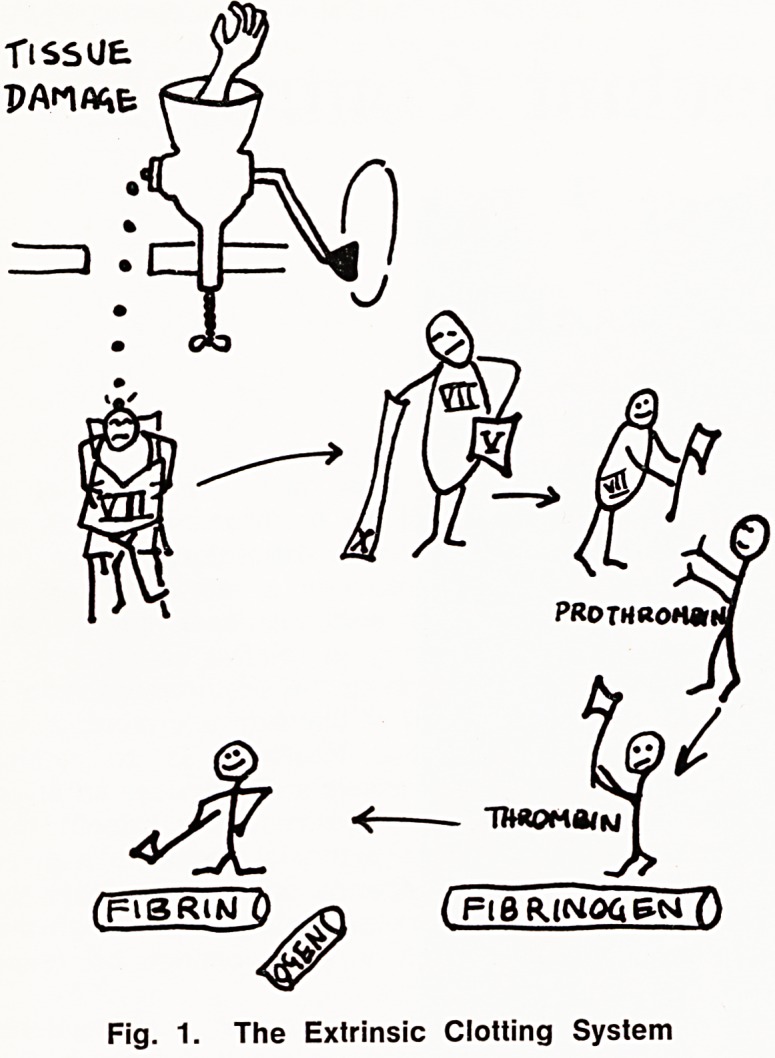


**Fig. 2. f2:**
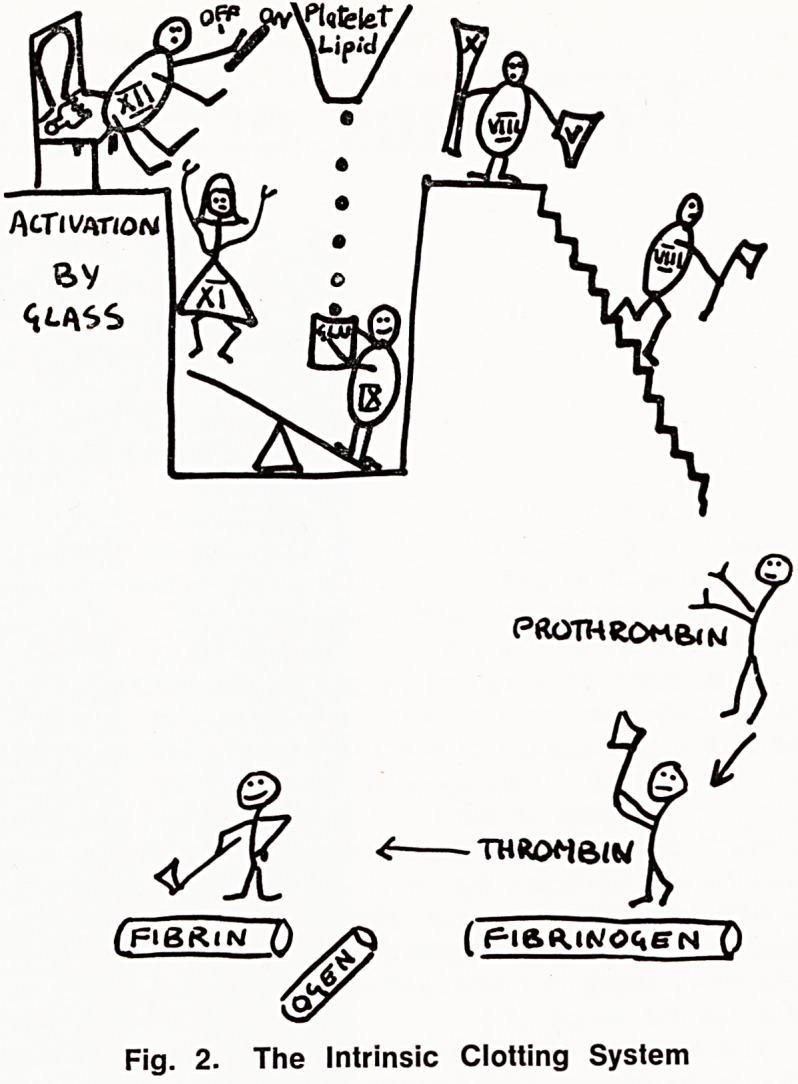


**Fig. 3. f3:**
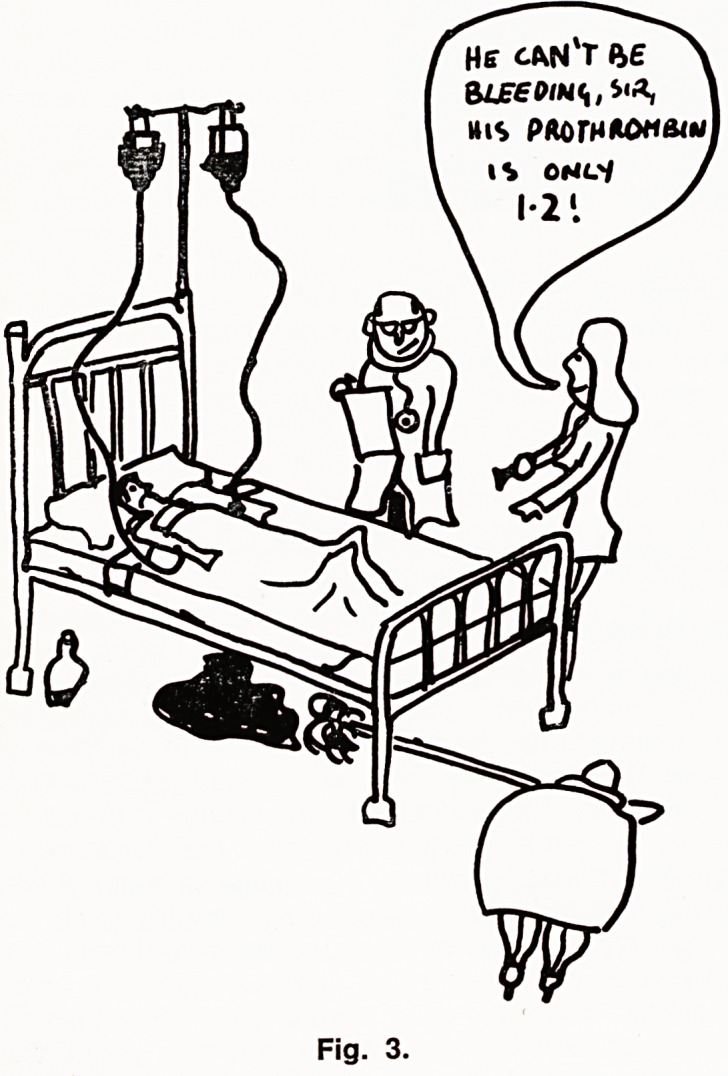


**Fig. 4. f4:**
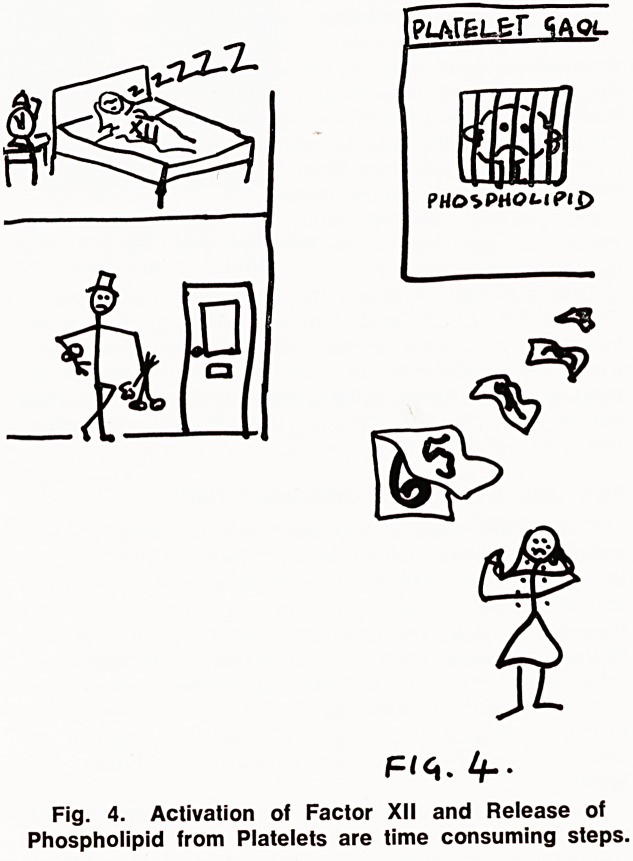


**Fig. 5. f5:**